# Solicited Cough Sound Analysis for Tuberculosis Triage Testing: The CODA TB DREAM Challenge Dataset

**DOI:** 10.1101/2024.03.27.24304980

**Published:** 2024-03-28

**Authors:** Sophie Huddart, Vijay Yadav, Solveig K. Sieberts, Larson Omberg, Mihaja Raberahona, Rivo Rakotoarivelo, Issa N. Lyimo, Omar Lweno, Devasahayam J Christopher, Nguyen Viet Nhung, Grant Theron, William Worodria, Charles Y. Yu, Christine M Bachman, Stephen Burkot, Puneet Dewan, Sourabh Kulhare, Peter M Small, Adithya Cattamanchi, Devan Jaganath, Simon Grandjean Lapierre

**Affiliations:** 1 University of California San Francisco, School of Medicine, 533 Parnassus Ave, San Francisco, CA 94143 USA; 2 Sage Bionetworks, Seattle, WA 98103 USA; 3 Curently at Koneksa Health, One World Trade Center 285 Fulton St. 77th Floor New York, NY, 10007; 4 CHU Joseph Rasera Befelatanana, Antananarivo, 101, Analamanga, Madagascar; 5 Centre d’Infectiologie Charles Mérieux, Antananarivo, 101, Analamanga, Madagascar; 6 CHU Tambohobe Fianarantsoa, 301, Haute-Matsiatra, Madagascar; 7 Université de Fianarantsoa, Fianarantsoa, 301, Haute-Matsiatra, Madagascar; 8 Ifakara Health Institute, Environmental and Ecological Sciences & Interventions and Clinical Trials Departments, Kiko Avenue, Plot 463, Mikocheni, Dar es Salaam, Tanzania; 9 Christian Medical College, Ida Scudder Road, Vellore 632004, Tamil Nadu, India; 10 National Tuberculosis Programme, 463 Hoang Hoa Tham, Ba Dinh District, Hanoi, Vietnam; 11 Stellenbosch University, Division of Molecular Biology and Human Genetics, Matieland, 7602 South Africa; 12 Walimu, Plot 5-7, Coral Crescent, Kololo, Kampala, Uganda; 13 De La Salle Medical and Health Sciences Institute, Governor D. Mangubat Avenue, Dasmarinas Cavite, Philippines 4114; 14 Global Health Labs, 14360 SE Eastgate Way, Bellevue, WA 98007 USA; 15 University of California Irvine, School of Medicine, 333 City Blvd. W Suite 400, Orange CA 92868 USA; 16 Centre de Recherche du Centre Hospitalier de l’Université de Montréal, Immunopathology Axis, 900 St-Denis, Montréal, Québec, H2X 0A9 Canada; 17 Université de Montréal, Department of Microbiology, Infectious Diseases and Immunology, 2900 Edouard-Montpetit, Montréal, Québec, H3T 1J4 Canada

## Abstract

Cough is a common and commonly ignored symptom of lung disease. Cough is often perceived as difficult to quantify, frequently self-limiting, and non-specific. However, cough has a central role in the clinical detection of many lung diseases including tuberculosis (TB), which remains the leading infectious disease killer worldwide. TB screening currently relies on self-reported cough which fails to meet the World Health Organization (WHO) accuracy targets for a TB triage test. Artificial intelligence (AI) models based on cough sound have been developed for several respiratory conditions, with limited work being done in TB. To support the development of an accurate, point-of-care cough-based triage tool for TB, we have compiled a large multi-country database of cough sounds from individuals being evaluated for TB. The dataset includes more than 700,000 cough sounds from 2,143 individuals with detailed demographic, clinical and microbiologic diagnostic information. We aim to empower researchers in the development of cough sound analysis models to improve TB diagnosis, where innovative approaches are critically needed to end this long-standing pandemic.

## BACKGROUND AND SUMMARY

Tuberculosis remains the leading infectious disease killer globally, partly due to public health systems’ inability to accurately diagnose millions of infected individuals every year.^1^ Insufficient access to high-quality TB screening and diagnosis is recognized as one of the most important gaps in the cascade of care.^2^ Here we describe a cough sound database including detailed demographic, clinical and microbiologic information for the development of AI-based sound classification TB triage models. As the WHO’s End TB Strategy calls for intensified research and innovation including the discovery of new tools for community-based screening, digital cough monitoring and *Acoustic Epidemiology* could represent new tools that can help bend the TB pandemic curve and accelerate the achievement of global TB elimination goals.^3–5^

The “missing millions” of undiagnosed patients living with active TB disease represent an heterogeneous group including those who did not access triage or diagnosis testing or weren’t appropriately referred for effective treatment. Improving the accuracy, portability, point-of-care amenability and connectivity of diagnostic tools and algorithms would have significant value. Most health systems build their TB programs on a combination of complementary screening followed by diagnostic tests. The WHO’s target product profile (TPP) for a community-based TB triage test suggests that it should be at least 90% sensitive and 70% specific.^6^ According to the 2021 WHO TB screening guidelines, symptom-based screening with questionnaires, including cough, is 42% sensitive.^7^ Besides having poor accuracy these guidelines have operational challenges that impede its sustained and uniform implementation within resource-challenged TB programs. Other tools such as digital chest X-rays combined with computer-aided detection (CAD) algorithms have also been evaluated in the context of TB triage. This approach was shown to be highly sensitive but had variable specificity and remains difficult to deploy due to limited availability of chest X-ray platforms at primary-level health facilities.^8^ Whether in the context of community-based outreach screening or healthcare facility-based evaluation prior to confirmatory testing, cough classification models could complement or replace other triage strategies including symptom-based screening.

We historically have been unable to objectively monitor cough sounds and consequently reduced this data-rich symptom into subjective and dichotomous information (e.g., cough versus no cough, chronic versus acute, better versus worse). Advances in acoustics and machine learning (ML) have enabled the identification and recording of human coughs in real-world acoustic environments (cough detection) as well as differentiation of coughs from patients with distinct clinical conditions or at different stages of disease (cough classification). As part of the emerging field of *Acoustic Epidemiology,* this has the potential to develop novel screening or diagnostic assays with simple digital recording devices, such as a smartphone, tablet or watch.^5^ Proof-of-concept studies previously showed that cough associated with TB contains a specific acoustic signature which can be recognized by ML models. A study by *Pahar et al*. suggests that a cough-based TB screening model can discriminate TB cough sounds from those associated with other lung conditions with 93% sensitivity and 95% specificity, exceeding the WHO TPPs.^9^ In a study combining cough sound analysis and patients’ clinical characteristics, *Yellapu et al.* report that ML can be used to detect TB with 90% sensitivity and 85% specificity.^10^ Those pilot studies report on ML models which were designed on small datasets and were not validated in external populations. Given the potential impact on performance of local disease epidemiology and population ethnicity among other confounders, large and diverse cough datasets are needed to replicate those studies.

We collected and are here releasing a dataset including 733,756 cough sounds from 2,143 patients across 7 countries with accurately annotated demographic, clinical and microbiologic diagnostic information. These data were initially used to enable and evaluate the CODA TB DREAM Challenge which invited participants to develop algorithms for prediction of TB diagnosis. The training data are now available for general use, and researchers are invited to leverage acoustic and clinical data to further develop and evaluate sound classification models for TB screening against a held-out test partition.^11^ We aim to enable the development of models which could achieve the WHO TPP performance targets for the current ‘community-based TB triage test’ or the forthcoming TPP for a TB screening test.^6,12^ This data set has limitations which include some selection bias since it was collected from a symptomatic presumptive TB population. The developed models which will be developed may hence not perform as well if used for asymptomatic screening at population level. Accordingly, more data should be collected from community screening activities.

## METHODS

### Participants

A total of 2,143 participants were recruited from two parent studies described below. To be eligible, participants had to be 18 years or older and have a new or worsening cough for at least two weeks. took place at outpatient clinics in India, Madagascar, the Philippines, South Africa, Tanzania, Uganda, and Vietnam. All participants provided informed consent. A summary of participant demographics and country distribution are available in Table 1.

#### Rapid Research in Diagnostic Development TB Network (R2D2 TB Network) study:

The R2D2 TB Network study evaluates novel TB diagnostics in various stages of development among people with presumptive TB in five low- and middle-income countries: Uganda, South Africa, Vietnam, the Philippines and India.^13^ Ethical approval for this study was obtained from institutional review boards (IRB) in the US and in each study site. In the US, approval was obtained from the University of California San Francisco IRB (# 20–32670). In Vietnam, approval was obtained from the Ministry of Health Ethical Committee for National Biological Medical Research (94/CN-HĐĐĐ), the National Lung Hospital Ethical Committee for Biological Medical Research (566/2020/NCKH) and the Hanoi Department of Health, Hanoi Lung Hospital Science and Technology Initiative Committee (22/BVPHN). In India, approval was obtained from Christian Medical College IRB (13256). In South Africa, approval was obtained from Stellenbosch University Health Research Ethics Committee (17047). In Uganda, approval was obtained from Makerere University, College of Health Sciences, School of Medicine, Research Ethics Committee (2020–182). In the Philippines, approval was obtained from De La Salle Health Sciences Institute Independent Ethics Committee (2020–33-02-A).

#### The Digital Cough Monitoring for screening, diagnosis and clinical follow-up of tuberculosis and other respiratory diseases project:

This project was designed to embed digital cough monitoring within existing health facility-based TB diagnostic cohorts in Madagascar and Tanzania. Ethical approval for this study was obtained from institutional review boards (IRB) in Canada and in each study site. In Canada, approval was obtained from the Centre de Recherche du Centre Hospitalier de l’Université de Montréal IRB (# 2021–9270, 20.226). In Madagascar, approval was obtained from the Comité d’Éthique à la Recherche Biomédicale (IORG0000851 - N°051-MSANP/SG/AMM/CERBM).). In Tanzania, approval was obtained from the Ifakarah Health Institute IRB (31–2021) and the National Institute for Medical Research (NIMR/HQ/R.8a/Vol IX/3805).

### Data Collection

#### Demographic and clinical data.

At enrollment into the parent studies, participants underwent a baseline questionnaire, clinical examination, and sputum collection for TB testing. Study staff also recorded participants’ age, gender, height, weight, smoking status and duration of cough. HIV diagnosis was made either based on participant self-report of a positive HIV diagnosis or a positive test result. A summary of the available variables is shown in Table 2.

#### TB Reference Standard Testing.

Both Xpert MTB/RIF Ultra PCR and mycobacterial culture (Lowenstein-Jensen solid medium or MGIT liquid medium) were performed on sputum collected from all participants. Any participant whose first sputum Xpert MTB/RIF Ultra result was indeterminate or trace-positive, received a second sputum Xpert MTB/RIF Ultra test. Results from those assays were combined to determine TB status according to two reference standards: a microbiologic reference standard and a sputum Xpert reference standard. The sputum Xpert reference standard is restricted to Xpert MTB/RIF Ultra results on sputum samples. The microbiologic reference standard includes culture results, allowing for more individuals to be classified as TB positive. The microbiologic reference standard is considered the primary reference standard. Full details of the reference standards are described in Table 3.

#### Cough Recording.

Cough sounds were collected using smartphones loaded with the Hyfe research app.^14^ Specific phone models used in the different participating sites are presented in Supplementary Materials 1. Hyfe research app is designed to listen for explosive sounds and record ~0.5 seconds sound fragments corresponding to putative cough sounds. Hyfe research app uses a server-based convolutional neural network (CNN) model to classify explosive sounds as coughs and recordings of these cough sounds are saved on a protected health information (PHI)-regulated server for analysis. This model has been shown to be 96% sensitive and 96% specific for cough detection using human-labeled sounds as a reference standard.^15^ Smartphones were positioned on tripods in rooms within the clinic. Participants were asked to cough five times (solicited cough) while standing 60–90 cm from the tripod; participants who managed to produce at least three coughs were retained in the dataset. Some participants produced more than five coughs due to a triggered coughing fit and those additional coughs were also collected and included in the dataset. Solicited and triggered coughs could not be labeled distinctively and are treated the same in the dataset. After enrollment and onboarding, a subset of participants (n = 565) were also asked to carry a study phone for two weeks and collect longitudinal coughs sounds in an outpatient setting. Those sounds are labeled as longitudinal and made available within the dataset. A tally of solicited and longitudinal cough sounds per data partition are available in Table 4.

### Data Partitioning

The dataset was split into a training (n=1,105) and validation set (n=1,038). The dataset was randomly partitioned evenly between the training and testing set at the level of the participant (i.e., all of a participant’s cough sounds are in either the training or validation set).

### Data Pre-Processing

#### Cough sounds:

The sound recordings available in this dataset have not undergone pre-processing beyond their identification as a cough sound by the Hyfe research app CNN model.

#### Clinical Data:

Data from all participating sites were collected with standardized questionnaires and definitions. Data formatting was harmonized in the open access database.

### Dataset Description

Sage Bionetworks independently verified the variable balance between the training and validation sets as demonstrated in Table 1. A breakdown of key demographics and microbiologic reference standard results by country are shown in Table 5.

## DATA RECORDS

De-identified participant demographic and clinical data, including TB reference standard results, cough sound WAV files, and a datafile linking participant IDs to sound file IDs were exported to a dedicated project in Synapse. Synapse is a general-purpose data and analysis sharing service where members can work collaboratively, analyze data, share insights, and have attributions and provenance of those insights to share with others. Synapse is developed and operated by Sage Bionetworks^16^. A total of 1,105 participants’ data are made available for access and download as a training dataset. The validation set is withheld, but models can be evaluated against the validation set via the instructions provided in the Synapse project.

All training set files are stored and are accessible via the Synapse platform with associated metadata and documentation and can be accessed at the following URL: www.synapse.org/TBcough - https://doi.org/10.7303/syn31472953.

## TECHNICAL VALIDATION

All cough collection periods were observed by study staff and cough sounds were spot-checked for accurate recording. Patient metadata was reviewed by study staff for accuracy. The data described in this article were collected using the Hyfe Research app which uses a proprietary algorithm to identify cough sounds. We used a prediction score of 0.8 from this algorithm to filter potential non-cough sounds. To validate the precision of the Hyfe algorithm, a standalone computer vision and deep learning model was trained using Log Mel spectrogram images from ESC-50 and Coswara datasets.^17,18^ The “VGG16” CNN based pre-trained model was trained for accurate classification of cough sounds and achieved a model accuracy of approximately 96% on Hyfe cough recordings.^19^ Most of the recordings that were classified incorrectly had a Hyfe prediction score less than 0.8.

## USAGE NOTES

Users can register to evaluate predictive models of TB diagnosis against the held-out test partition via the instructions on the Synapse project. The scoring mechanism can evaluate two different types of models: (1) those that use only cough sounds, or (2) those which also incorporate clinical metadata variables which have been provided in the training dataset (sex, age, height, weight, reported duration of cough, prior TB diagnosis and type, hemoptysis, heart rate, temperature, weight loss, smoking in the last week, fever and night sweats). Models are submitted to the scoring queues as Docker images. Full instructions and example code is available on Synapse project website (www.synapse.org/TBcough).

### Downloading the Data

Given the number of files represented in the data, users should consider downloading the data via one of the programmatic Synapse clients (available in R or Python). For convenience, Python code for downloading the data is provided in the Synapse project wiki. The training dataset size is (0.43 GB) for the solicited coughs and (31.6 GB) for the longitudinal coughs.

### Data Use Agreement

To access the data, individuals must become Certified and Validated users of Synapse and maintain an active account on Synapse: http://www.synapse.org. They must also submit an Intended Data Use Statement and agree to the Terms of Use of the dataset. Terms of Use are summarized in Supplementary Materials 2.

## Supplementary Material

Supplement 1

## Figures and Tables

**Table 1 –:** Participant demographics across training and test sets

	Training set (N=1105)	Testing set (N=1038)	Complete set (N=2143)

**Sex**			

Female	517 (46.8%)	460 (44.3%)	977 (45.6%)

Male	588 (53.2%)	578 (55.7%)	1166 (54.4%)

**Age**			
Median [Q1, Q3]	40.0 [28, 53]	40.0 [29, 53]	40.0 [28, 53]

**Height (cm)**			
Median [Q1, Q3]	162 [155, 168]	162 [156, 168]	162 [156, 168]
**Weight (Kg)**			

Median [Q1, Q3]	55.0 [49, 65]	56.0 [49, 65]	55.8 [49, 65]

**HIV status**			

Negative	878 (79.5%)	809 (77.9%)	1687 (78.7%)

Positive	162 (14.7%)	155 (14.9%)	317 (14.8%)

Unknown	65 (5.9%)	74 (7.1%)	139 (6.5%)

**Duration of cough (days)**			
Median [Q1, Q3]	30.0 [16, 60]	30.0 [16, 50]	30.0 [16, 60]

**Prior TB**			
No	903 (81.7%)	835 (80.4%)	1738 (81.1%)

Not sure	3 (0.3%)	2 (0.2%)	5 (0.2%)
Yes	199 (18.0%)	201 (19.4%)	400 (18.7%)

**Country**			
India	119 (10.8%)	122 (11.8%)	241 (11.2%)

Madagascar	159 (14.4%)	75 (7.2%)	234 (10.9%)

The Philippines	198 (17.9%)	190 (18.3%)	388 (18.1%)

South Africa	137 (12.4%)	138 (13.3%)	275 (12.8%)

Tanzania	87 (7.9%)	110 (10.6%)	197 (9.2%)

Uganda	242 (21.9%)	245 (23.6%)	487 (22.7%)

Vietnam	163 (14.8%)	158 (15.2%)	321 (15.0%)

**Microbiologic reference standard**			

TB Negative	807 (73.0%)	782 (75.3%)	1589 (74.1%)

TB Positive	297 (26.9%)	256 (24.7%)	553 (25.8%)
**Sputum Xpert reference standard**			

Indeterminate	4 (0.4%)	2 (0.2%)	6 (0.3%)
TB Negative	839 (75.9%)	808 (77.8%)	1647 (76.9%)

TB Positive	262 (23.7%)	226 (21.8%)	488 (22.8%)

**Table 2 -:** Available demographic, clinical and microbiologic variables

Variable	Values	Format/Definition
Country	Philippines Vietnam South Africa Uganda India Madagascar Tanzania	Country where the participant was enrolled
Sex	Male Female	Sex at birth reported by participant
Age	Numeric	Age calculated as date of collection - date of birth if known. If date of birth is unknown, reported age at time of collection.
Height	Numeric	Height in centimeters
Weight	Numeric	Weight in Kg
HIV status	Positive Negative	HIV status. All participants who do not report being HIV-positive receive HIV testing (using capillary or venous blood) Positive = Positive reported by patient or positive on a HIV test Negative = Negative on a HIV test
Reported duration of cough	Numeric	Self-reported duration of current cough (days). At baseline, we ask: How many days have you had this new cough or cough that has been worse?
Prior TB	Yes No	Self-reported. At baseline, we ask: Have you ever had or been told you had tuberculosis (TB)?
Prior TB type: Pulmonary	Checked Unchecked	Asked at baseline: “With what kind of TB were you diagnosed?” (may select more than one) Participant selected pulmonary TB.
Prior TB type: Extrapulmonary	Checked Unchecked	Asked at baseline: “With what kind of TB were you diagnosed?” (may select more than one) Participant selected extrapulmonary TB.
Prior TB type: Unknown	Checked Unchecked	Asked at baseline: “With what kind of TB were you diagnosed?” (may select more than one) Participant selected Unknown.
Hemoptysis	Yes No	In the past 30 days, have you ever coughed up blood?
Heart rate	Numeric	Participanťs heartrate (beats per minute) measured at baseline
Temperature	Numeric	Participanťs temperature (Celsius) measured at baseline.
Weight loss	Yes No	Subjective, Self-reported. At baseline, we ask: In the past 30 days, have you experienced any weight loss?
Smoked in last week	Yes No	Asked at baseline “Have you used combustible tobacco and/or vaping products in the last 7 days?”
Fever	Yes No	Self-reported fever at baseline: “In the past 30 days, have you ever felt or experienced fever?”
Night sweats	Yes No	Self-reported night sweats at baseline: “In the past 30 days, have you ever experienced night sweats?”
Microbiologic reference standard*	TB Positive TB Negative Indeterminate	TB diagnosis based on sputum and culture results
Sputum Xpert reference standard*	Positive Negative Indeterminate	TB diagnosis based on sputum results alone
Xpert combined semi-quant	Trace Very Low Low Medium High	Highest semiquantitative result from sputum Xpert Ultra test conducted at baseline visit. A semiquantitative result will only be available if the test was positive.

**Table 3 –:** Reference standard definitions

Result	Microbiologic Reference Standard	Sputum Xpert Reference Standard
TB positive	One or more of the following: • a positive sputum Xpert result • a positive urine Xpert result • a positive liquid culture result • a positive solid culture result • a positive Xpert Ultra results on contaminated liquid culture • two trace positive Xpert Ultra results on any sample type.	One of the following: • a positive sputum Xpert result • two trace-positive sputum Xpert Ultra results
TB negative	No positive results on any Xpert or Xpert Ultra testing and one of the following conditions: • two negative liquid cultures • two negative solid cultures • one negative liquid and one negative solid cultures	A negative sputum Xpert result
Indeterminate	No positive results on any Xpert or Xpert Ultra testing and only one trace result on Xpert Ultra testing of any sample type	An indeterminate or trace-positive sputum Xpert result followed by a non-positive result on repeat sputum Xpert Ultra testing

**Table 4 –:** Number of cough recordings per data partition

	Training set	Testing set
Solicited coughs (Participants)	9,772 (1,082)	9,062 (1,038)
Longitudinal coughs (participants)	714,922 (565)	0 (0)

Longitudinal coughs are not used for evaluation on the testing set, so are not quantified here.

**Table 5 –:** Key variables summarized by country

	India n = 241	Madagascar n = 234	Philippines n = 388	South Africa n = 275	Tanzania n = 197	Uganda n = 487	Vietnam n = 321
**Sex**							
Female	100 (41%)	115 (49%)	208 (54%)	144 (52%)	86 (44%)	203 (42%)	121 (38%)
Male	141 (59%)	119 (51%)	180 (46%)	131 (48%)	111 (56%)	284 (58%)	200 (62%)
**Age**							
Median [Q1, Q3]	47 [34, 58]	32 [24, 51]	39 [26, 53]	40 [32, 49]	40 [31,50]	32 [26, 42]	54 [41,64]
**Duration of cough (days)**							
Median [Q1, Q3]	45 [30, 90]	30 [17, 60]	21 [15, 30]	21 [14, 28]	30 [14, 30]	30 [16, 60]	30 [30, 90]
**Prior TB**							
No	204 (85%)	207 (88%)	325 (84%)	170 (62%)	147 (75%)	427 (88%)	258 (80%)
Not sure	0 (0%)	0 (0%)	4 (1.0%)	0 (0%)	0 (0%)	0 (0%)	1 (0.3%)
Yes	37 (15%)	27 (12%)	59 (15%)	105 (38%)	50 (25%)	60 (12%)	62 (19%)
**Microbiologic reference standard**							
TB Negative	216 (90%)	131 (56%)	348 (90%)	223 (81%)	161 (82%)	313 (64%)	199 (62%)
TB Positive	25 (10%)	103 (44%)	40 (10%)	52 (19%)	36 (18%)	174 (36%)	122 (38%)

## Data Availability

All training set files are stored and are accessible via the Synapse platform with associated metadata and documentation and can be accessed at the following URL: www.synapse.org/TBcough. Specific acoustic and clinical metadata as well as dataset information can be found under the following doi references. Clinical data: https://doi.org/10.7303/syn53710097 Cough metadata: https://doi.org/10.7303/syn53710098 Solicited Coughs: https://doi.org/10.7303/syn40358494 Longitudinal Coughs: https://doi.org/10.7303/syn40358476 Data Dictionary: https://doi.org/10.7303/syn41743692 Data sharing and model benchmarking website: https://doi.org/10.7303/syn31472953
