# Supplementary material for: Solicited Cough Sound Analysis for Tuberculosis Triage Testing: The CODA TB DREAM Challenge Dataset
Source: medRxiv. 2024 Mar 28:2024.03.27.24304980. Preprint. [Version 1] doi: 10.1101/2024.03.27.24304980 (PMC10996751; doi:10.1101/2024.03.27.24304980)
Supplement: Supplement 1 [file NIHPP2024.03.27.24304980v1-supplement-1.pdf]

medRxiv preprint doi: https://doi.org/10.1101/2024.03.27.24304980; this version posted March 28, 2024. The copyright holder for this preprint (which was not certified by peer review) is the author/funder, who has granted medRxiv a license to display the preprint in perpetuity.
It is made available under a CC-BY 4.0 International license .

SUPPLEMENTARY MATERIALS

Supplementary materials 1 - Phone models used in the different participating sites

India

- Redmi 9 Prime
- Realme Narzo20

Madagascar

- Motorola G9 play

Philippines

- Myphone myWX2 Pro
- Xiaomi 9C

South Africa

- Nokia 3.1
- Nokia 5.4
- Xiaomi Redmi 9A

Tanzania

- Nokia 3.4 Ta-1288

Uganda

- Motorola G16
- Samsung M11
- Nokia model 5.3

Vietnam

- OPPOA54

medRxiv preprint doi: https://doi.org/10.1101/2024.03.27.24304980; this version posted March 28, 2024. The copyright holder for this preprint (which was not certified by peer review) is the author/funder, who has granted medRxiv a license to display the preprint in perpetuity.
It is made available under a CC-BY 4.0 International license .

Supplementary materials 2 – Data Use Agreement

Researchers wishing to access the data must:

- You must reaffirm your commitment to the Synapse Pledge and must abide by the guiding principles for responsible research use and data handling within the Synapse Commons Platform as described in the Synapse Governance documents.
- You will not attempt to establish the identity of, or attempt to contact any of the subjects included in the data.
- You confirm that if you inadvertently receive identifiable information or otherwise identify a subject, you will promptly notify the ACT by emailing act@sagebase.org.
- You agree to establish appropriate administrative, technical, and physical safeguards to prevent unauthorized use of or access to the Data.
- You will report any data misuse or breach of data security to ACT by emailing act@sagebase.org.
- You will use the data only as identified in your intended data use statement (IDU), submitted through Synapse. The IDU should be written in English and must describe the objectives of the proposed research and study design and analysis plan (500 word maximum).
- Data accessors must acknowledge the following in all publications or presentations as follows:
  "The datasets used for the analyses described were contributed by Dr. Adithya Cattamanchi at UCSF and Dr. Simon Grandjean Lapierre at University of Montreal and were generated in collaboration with researchers at Stellenbosch University (PI Grant Theron), Walimu (PIs William Worodria and Alfred Andama); De La Salle Medical and Health Sciences Institute (PI Charles Yu), Vietnam National Tuberculosis Program (PI Nguyen Viet Nhung), Christian Medical College (PI DJ Christopher), Centre Infectiologie Charles Mérieux Madagascar (PIs Mihaja Raberahona & Rivonirina Rakotoarivelo), and Ifakara Health Institute (PIs Issa Lyimo & Omar Lweno) with funding from the U.S. National Institutes of

medRxiv preprint doi: https://doi.org/10.1101/2024.03.27.24304980; this version posted March 28, 2024. The copyright holder for this preprint (which was not certified by peer review) is the author/funder, who has granted medRxiv a license to display the preprint in perpetuity.
It is made available under a CC-BY 4.0 International license .

Health (U01 AI152087), The Patrick J. McGovern Foundation and Global Health Labs.”
